# Characteristics of Patients with Acute Flaccid Myelitis, United States, 2015–2018

**DOI:** 10.3201/eid2602.191453

**Published:** 2020-02

**Authors:** Nilay McLaren, Adriana Lopez, Sarah Kidd, John Zhang, W. Allan Nix, Ruth Link-Gelles, Adria Lee, Janell A. Routh

**Affiliations:** Centers for Disease Control and Prevention, Atlanta, Georgia, USA

**Keywords:** myelitis, paralysis, enterovirus, pediatrics, acute flaccid myelitis, United States, clinical characteristics, demographics, epidemiology, pleocytosis, viruses, surveillance, EV-D68, respiratory infections

## Abstract

Observed peaks of acute flaccid myelitis (AFM) cases have occurred biennially since 2014 in the United States. We aimed to determine if AFM etiology differed between peak and nonpeak years, considering that clinical features of AFM differ by virus etiology. We compared clinical and laboratory characteristics of AFM cases that occurred during peak (2016 and 2018, n = 366) and nonpeak (2015 and 2017, n = 50) years. AFM patients in peak years were younger (5.2 years) than those in nonpeak years (8.3 years). A higher percentage of patients in peak years than nonpeak years had pleocytosis (86% vs. 60%), upper extremity involvement (33% vs. 16%), and an illness preceding limb weakness (90% vs. 62%) and were positive for enterovirus or rhinovirus RNA (38% vs. 16%). Enterovirus D68 infection was associated with AFM only in peak years. Our findings suggest AFM etiology differs between peak and nonpeak years.

## Abstract 2

Differences between years with and without increased activity suggest differences in viral etiologies.

Acute flaccid myelitis (AFM) is a clinical syndrome characterized by the acute onset of flaccid limb weakness accompanied by spinal cord gray matter lesions. AFM is a known complication of infection with certain viruses, including polioviruses, nonpolio enteroviruses, flaviviruses, herpesviruses, and adenoviruses (*1*–*7*). In the early 1950s, outbreaks of poliovirus caused >15,000 cases of paralysis each year in the United States, but after the introduction of poliovirus vaccines and the elimination of poliovirus in the United States, AFM caused by poliovirus became much less common (*8*). However, sporadic, poliovirus-negative cases continued to occur (*9*).

In August 2014, an unusual cluster of AFM in children was identified in Colorado (*10*). National surveillance was initiated in 2015 and subsequently led to the identification of heightened activity in 2016 and 2018; during these years, peak illness onset occurred during August–October. In contrast, in 2015 and 2017, the number of AFM cases remained low and did not vary by season (*11*). National experts agree that the AFM epidemiology observed in 2014 is new; this alternating pattern of high activity one year and low activity the next, referred to herein as peak and nonpeak years, with high activity typically in the late summer or early fall, has not been documented before 2014 (*12*–*14*) (M. Cortese, Centers for Disease Control and Prevention [CDC], Atlanta, GA, USA, pers. comm., 2017 Sep 27). This change suggests the emergence (beginning in 2014) of either a new cause of AFM or a known cause of AFM with a new epidemiologic pattern.

Determining the cause or causes of the biennial increases in AFM cases has implications for the development of treatment and prevention strategies. However, pathogen-specific laboratory testing has yielded limited insight into the underlying cause of this new epidemiology. Different viruses known to be associated with AFM development have been shown to produce distinctive sets of clinical features (*15*,*16*). If a single pathogen is responsible for most AFM cases in peak years, cases of illness onset in these years probably would have clinical manifestations distinct from those of illness onset in nonpeak years, when the etiology of cases is likely more mixed. We compared demographic, clinical, and laboratory characteristics of AFM cases in peak versus nonpeak years to evaluate this hypothesis.

## Methods

### Reporting and Classification

Beginning in August 2014, CDC received reports of patients meeting the clinical criterion for AFM (i.e., acute onset of flaccid limb weakness) through local and state health departments. A panel of expert neurologists classified these patients according to the standardized case definition published by the Council of State and Territorial Epidemiologists in 2015 (*17*). We defined a confirmed case as an illness in a patient who met the clinical criterion and had magnetic resonance imaging (MRI) showing a spinal cord lesion largely restricted to the gray matter and spanning >1 spinal segment. Our analysis includes only confirmed AFM cases in patients <22 years of age and is limited to 4 complete years of AFM surveillance (January 1, 2015–December 31, 2018).

### Laboratory Testing

CDC staff requested sterile site (e.g., blood, serum, and cerebrospinal fluid [CSF]) and nonsterile site (e.g., respiratory and fecal) specimens from each patient and tested these specimens using algorithms described previously (*18*,*19*). With specimens from 2015 and 2016, and starting in September 2018 with all received specimens, CDC staff tested for enterovirus/rhinovirus RNA using a 5′ nontranslated region–targeted pan-*Enterovirus* real-time reverse transcription PCR assay (genus-level detection) and typed those that were positive. For specimens collected during January 2017–August 2018, only the specimens that had tested positive for enterovirus/rhinovirus RNA at an outside institution were requested by CDC staff for testing and typing. In our analysis, we report only the results from CDC laboratory testing.

### Data Analysis

To assess trends in AFM activity over time, we assigned patients with confirmed cases to an epidemiologic week according to their date of onset of limb weakness. We compared cases of patients having AFM onset in peak years (i.e., 2016 and 2018) with those of patients having AFM onset in nonpeak years (i.e., 2015 and 2017) and compared cases between the 2 peak years (2016 vs. 2018). We analyzed the demographics, clinical characteristics, and laboratory results of AFM patients that had been systematically collected across all 4 years of surveillance. We defined AFM cases as severe if they included all 3 of the following clinical characteristics: respiratory distress requiring mechanical ventilation to manage, symptomatic cranial nerve involvement, and paralysis of all 4 limbs. We defined CSF pleocytosis as a leukocyte count of >5 cells/mm^3^.

We entered data into a Microsoft Access (for 2015–2017 data; https://www.microsoft.com) or REDCap (for 2018 data; https://www.project-redcap.org) database and performed descriptive analyses using R Studio version 3.4.1 (https://rstudio.com). Denominators varied slightly by variable because of missing data. We assessed differences in categorical variables using Fisher exact test and compared medians using the Kruskal-Wallis test. We considered p values <0.05 statistically significant.

CDC staff determined that we collected data through the standardized public health surveillance system and not through research involving humans. Thus, this study did not require institutional review board clearance.

## Results

Of 750 suspected AFM cases reported to the CDC during 2015–2018, a total of 416 (n = 18 in 2015, n = 143 in 2016, n = 32 in 2017, and n = 223 in 2018) occurred in patients <22 years of age and were classified as confirmed. Cases in patients of this age group represented 95% of all confirmed cases. The median age of patients with confirmed cases was 5.4 (range 0.3–21.9, interquartile range 3.2–8.7) years; 60% were male. In peak years (2016 and 2018), the increase in confirmed AFM cases started in August, and for both peak years, most patients with confirmed cases had illness onset during August–October (Figure).

**Figure:**
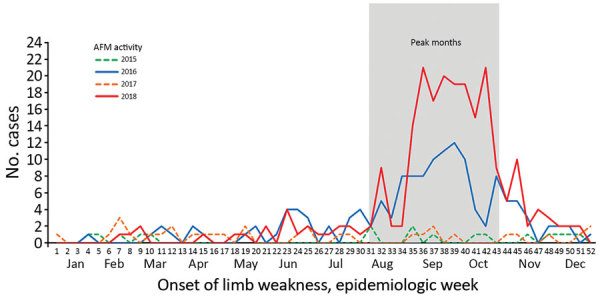
Confirmed AFM cases in patients <22 years of age by week of limb weakness onset, United States, January 2015–December 2018. AFM, acute flaccid myelitis.

When comparing the characteristics of confirmed AFM cases from peak years (2016 and 2018) and nonpeak years (2015 and 2017), we found that patient median age was significantly lower in peak years (5.2 [range 0.4–21.9] years of age) than nonpeak years (8.3 [range 0.3–20.2] years of age; p = 0.02) (Table 1). The limbs affected by AFM also varied; during peak years, a higher percentage of cases involved upper extremity weakness only (33% vs. 16%; p = 0.01) and a lower percentage involved lower extremity weakness only (13% vs. 32%; p<0.001). During peak years, fewer cases could be classified as severe (2% vs. 18%; p<0.001). The percentage of AFM patients who had a preceding illness (i.e., any fever or respiratory illness) during the 4 weeks before limb weakness onset was higher in peak years (90%) than nonpeak years (62%; p<0.001), and CSF pleocytosis was more common among AFM patients in peak years (86%) than in nonpeak years (60%; p<0.001). The percentage of patients with a specimen positive for enterovirus/rhinovirus RNA was significantly greater in peak years (38%) than nonpeak years (16%; p = 0.02). During peak years, a greater percentage of enterovirus/rhinovirus-positive specimens was positive for enterovirus D68 (EV-D68) RNA (54% vs. 0%; p = 0.02).

**Table 1:** Descriptive characteristics of confirmed AFM cases in patients <22 years of age from peak years versus nonpeak years, United States, January 2015–December 2018*

Characteristic	Peak years, n = 366†	Nonpeak years, n = 50‡	p value
Age, y, median (range) [IQR]	5.2 (0.4–21.9) [3.2–8.0]	8.3 (0.3–20.2) [3.9–11.9]	0.02
Sex			
M	217/366 (59)	32/50 (64)	0.54
F	149/366 (41)	18/50 (36)	0.54
Lumbar puncture	343/363 (94)	48/49 (98)	0.84
Pleocytosis§	283/328 (86)	28/47 (60)	<0.001
CSF, cells/mm^3^, median (range) [IQR]	75 (0–3,261) [24–150]	32 (0–1,081) [3–168]	0.07
Upper extremity involvement only	121/366 (33)	8/50 (16)	0.01
Lower extremity involvement only	49/366 (13)	16/50 (32)	<0.001
Paralysis of all 4 limbs	101/366 (28)	24/50 (48)	0.005
Severe AFM¶	9/366 (2)	9/50 (18)	<0.001
Cranial nerve lesion	96/366 (26)	10/50 (20)	0.39
Illness within previous 4 weeks			
Any fever	258/358 (72)	27/49 (55)	0.02
Any respiratory illness	282/360 (78)	21/49 (43)	<0.001
Any gastrointestinal illness	111/344 (32)	12/34 (35)	0.24
Any respiratory illness or fever	328/366 (90)	31/50 (62)	<0.001
Enterovirus/rhinovirus RNA positive	98/258 (38)	5/31 (16)	0.02
Enterovirus D68	53/98 (54)	0/5	0.02
Enterovirus A71	13/98 (13)	1/5 (20)	0.59

*Values are no./total (%), except where indicated otherwise. AFM, acute flaccid myelitis; CSF, cerebrospinal fluid; IQR, interquartile range. †2016 and 2018. ‡2015 and 2017. §Defined as leukocyte count >5 cells/mm^3^. ¶Defined as AFM cases of respiratory distress that required mechanical ventilation, cranial nerve involvement, and paralysis of all 4 limbs.

To evaluate whether the characteristics of cases from nonpeak months were masking the characteristics of cases from peak months, which we hypothesized to be associated with a single pathogen, we conducted a sensitivity analysis comparing cases in peak months (August–October) from peak years (2016 and 2018) with cases from all nonpeak months (January–July and November–December of 2016 and 2018, January–December of 2015 and 2017) (Table 2). Most variables remained significant in the sensitivity analysis. However, the difference in median age between peak and nonpeak years was no longer significant. The percentage of cases with pleocytosis remained significantly higher in peak months than nonpeak months (p<0.001), and in this analysis, the median cell count was also significantly higher in cases in peak months (88 cells/mm^3^) than in cases in nonpeak months (44 cells/mm^3^; p<0.001). The percentage of cases with a specimen positive for enterovirus or rhinovirus RNA was no longer significantly greater in peak months (38%) than nonpeak months (31%; p = 0.25). The percentage of EV-D68–positive cases was also no longer significantly greater in peak months (58%) than nonpeak months (37%; p = 0.08).

**Table 2:** Descriptive characteristics of confirmed AFM cases in patients <22 years of age from peak months versus nonpeak months, United States, January 2015–December 2018*

Characteristic	All peak months, n = 268†	All nonpeak months, n = 148‡	p value
Age, y, median (range) [IQR]	5.2 (0.5–21.9) [3.3–7.8]	5.7 (0.3−21.9) [3.2–10.6]	0.18
Sex			
M	159/268 (59)	90/148 (61)	0.83
F	109/268 (41)	58/148 (39)	0.83
Lumbar puncture	251/267 (94)	140/145 (97)	0.35
Pleocytosis§	212/239 (89)	99/136 (73)	<0.001
CSF, cells/mm^3^, median (range) [IQR]	88 (0–814) [30–154]	44 (0–3,261) [4–118]	<0.001
Upper extremity involvement only	95/268 (35)	34/148 (23)	0.01
Lower extremity involvement only	29/268 (11)	36/148 (24)	0.001
Paralysis of all 4 limbs	67/268 (25)	58/148 (39)	0.004
Severe AFM¶	5/268 (2)	13/148 (9)	0.002
Cranial nerve lesion	72/268 (27)	34/148 (23)	0.41
Illness within previous 4 weeks			
Any fever	201/262 (77)	84/145 (58)	<0.001
Any respiratory illness	214/264 (81)	89/145 (61)	<0.001
Any gastrointestinal illness	80/256 (31)	43/122 (35)	0.46
Any respiratory illness or fever	246/268 (92)	113/148 (76)	<0.001
Enterovirus/rhinovirus RNA positive	73/192 (38)	30/97 (31)	0.25
Enterovirus D68	42/73 (58)	11/30 (37)	0.08
Enterovirus A71	6/73 (8)	8/30 (27)	0.02

*Values are no./total (%), except where indicated otherwise. AFM, acute flaccid myelitis; CSF, cerebrospinal fluid; IQR, interquartile range. †August–October of 2016 and 2018. ‡January–July and November–December of 2016 and 2018, January–December of 2015 and 2017. §Defined as leukocyte count >5 cells/mm^3^. ¶Defined as AFM cases of respiratory distress that required mechanical ventilation, cranial nerve involvement, and paralysis of all 4 limbs.

Patients with illness onset in 2018 and illness onset in 2016 were clinically similar to each other (Table 3), with a few notable exceptions. Cranial nerve lesions were less common in AFM patients in 2018 (19%) than in AFM patients in 2016 (37%; p<0.001). More cases in 2016 (6%) than 2018 (0%; p<0.001) were classified as severe AFM. Compared with AFM patients in 2016, more AFM patients in 2018 were reported to have an illness within the 4 weeks preceding the onset of limb weakness: any fever (68% in 2016 vs. 75% in 2018; p<0.001), respiratory illness (76% in 2016 vs. 80% in 2018; p = 0.01), or gastrointestinal illness (26% in 2016 vs. 36% in 2018; p = 0.002). The percentage of confirmed AFM cases positive for enterovirus/rhinovirus RNA was similar in both peak years. Among all enterovirus/rhinovirus-positive specimens, EV-D68–positive specimens were more common in 2016 (71%) than in 2018 (45%; p = 0.02). However, AFM patients in 2018 (17%) were more likely than those in 2016 (6%; p = 0.21) to be positive for EV-A71 because of a geographically limited outbreak of EV-A71 in Colorado.

**Table 3:** Descriptive characteristics of confirmed AFM cases in patients <22 years of age from peak years, United States, January–December 2016 and 2018*

Characteristic	2016, n = 143	2018, n = 223	p value
Age, y, median (range) [IQR]	5.3 (0.4–21.2) [3.2–9.7]	5.2 (0.5–21.9) [3.2–7.8]	0.43
Sex			
M	86/143 (60)	131/223 (59)	0.83
F	57/143 (40)	92/223 (41)	0.83
Lumbar puncture	135/140 (96)	208/223 (93)	0.14
Pleocytosis†	114/135 (84)	169/208 (81)	0.47
CSF, cells/mm^3^, median (range) [IQR]	77 (0–3,261) [18–150]	74 (0–814) [27–152]	0.22
Upper extremity involvement only	48/143 (34)	73/223 (33)	0.91
Lower extremity involvement only	25/143 (17)	24/223 (11)	0.08
Paralysis of all 4 limbs	45/143 (31)	56/223 (25)	0.19
Severe AFM‡	9/143 (6)	0/223	<0.001
Cranial nerve lesion	53/143 (37)	43/223 (19)	<0.001
Illness within previous 4 weeks			
Any fever	93/137 (68)	165/221 (75)	<0.001
Any respiratory illness	106/139 (76)	176/221 (80)	0.01
Any gastrointestinal illness	33/126 (26)	78/218 (36)	0.002
Any respiratory illness or fever	122/143 (85)	206/223 (92)	0.04
Enterovirus/rhinovirus RNA positive	34/94 (36)	64/164 (39)	0.69
Enterovirus D68	24/34 (71)	29/64 (45)	0.02
Enterovirus A71	2/34 (6)	11/64 (17)	0.21

*Values are no./total (%), except where indicated otherwise. AFM, acute flaccid myelitis; CSF, cerebrospinal fluid; IQR, interquartile range. †Defined as leukocyte count >5 cells/mm^3^. ‡Defined as AFM cases of respiratory distress that required mechanical ventilation, cranial nerve involvement, and paralysis of all 4 limbs.

To determine whether the differences in case characteristics between peak years could be explained by the EV-A71 outbreak, we conducted a subanalysis in which we removed all EV-A71–positive cases. Differences in clinical characteristics between years remained unchanged; however, the percentage of cases positive for EV-D68 was no longer significantly different (75% vs. 55%; p = 0.07) (Table 4).

**Table 4:** Descriptive characteristics of confirmed AFM cases in patients <22 years of age who were negative for enterovirus A71 during peak years, United States, January–December 2016 and 2018*

Characteristic	2016, n = 141	2018, n = 212	p value
Age, y, median (range) [IQR]	5.4 (0.4–21.2) [3.3–9.8]	5.3 (0.5–21.9) [3.4–7.8]	0.68
Sex			
M	86/141 (61)	120/212 (57)	0.44
F	55/141 (39)	92/212 (43)	0.44
Lumbar puncture	134/139 (96)	197/212 (93)	0.13
Pleocytosis†	113/132 (86)	158/184 (86)	1
CSF, cells/mm^3^, median (range) [IQR]	73 (0–3,261) [18–150]	73 (0–814) [25.5–149]	0.86
Upper extremity involvement only	48/141 (34)	71/212 (33)	1
Lower extremity involvement only	25/141 (18)	23/212 (11)	0.08
Paralysis of all 4 limbs	44/141 (31)	50/212 (24)	0.14
Severe AFM‡	9/141 (6)	0/212	<0.001
Cranial nerve lesion	52/141 (37)	42/212 (20)	<0.001
Illness within previous 4 weeks			
Any fever	91/135 (67)	154/210 (73)	<0.001
Any respiratory illness	104/137 (76)	172/210 (82)	0.004
Any gastrointestinal illness	32/124 (26)	72/207 (35)	0.003
Any respiratory illness or fever	120/141 (85)	195/212 (92)	0.05
Enterovirus/rhinovirus RNA positive	32/92 (35)	53/153 (35)	1
Enterovirus D68	24/32 (75)	29/53 (55)	0.07

*Values are no./total (%), except where indicated otherwise. AFM, acute flaccid myelitis; CSF, cerebrospinal fluid; IQR, interquartile range. †Defined as leukocyte count >5 cells/mm^3^. ‡Defined as AFM cases of respiratory distress that required mechanical ventilation, cranial nerve involvement, and paralysis of all 4 limbs.

## Discussion

Five years of national AFM surveillance data show a seasonal, alternate-year pattern of AFM activity. The numbers of confirmed cases in peak years (2016 and 2018) was >5 times the numbers of cases in the previous year. Our analysis demonstrates key clinical differences between cases in peak years and nonpeak years. Patients with AFM onset in peak years were younger, more likely to have had fever or respiratory symptoms within the 4 weeks preceding AFM onset, and more likely to have pleocytosis and upper extremity weakness during hospitalization than those with AFM onset in nonpeak years. These clinical characteristics mirror the presentation of AFM described for cases of onset in 2014 (*20*). Patients with onset in nonpeak years were more severely affected during acute illness than those with onset in peak years. We also found differences between AFM patients with onset in 2016 and AFM patients with onset in 2018, the 2 peak years. Compared with AFM patients with onset in 2016, those with onset in 2018 were less likely to have severe disease or cranial nerve lesions and were more likely to have a preceding illness before AFM onset.

Differences in the clinical presentation of AFM between peak and nonpeak years are suggestive of differences in AFM etiologies between those years. Likewise, the clinical differences between cases occurring during peak and nonpeak years might be indicative of different virus etiologies between peak and nonpeak years. In our analysis, enterovirus positivity among AFM cases was higher in peak years than in nonpeak years, and EV-D68 was detected only in the cases occurring during peak years. Although this difference disappeared in the sensitivity analysis (suggesting that the enteroviruses circulating in peak years varies slightly by month), EV-D68 detections remained exclusive to cases occurring in peak years. AFM case counts also vary slightly by month in different peak years; the escalation in cases began earlier in 2016 than they did in 2018, and the increase in case counts lasted slightly longer in 2018.

In peak years, overall enterovirus positivity among AFM cases was similar, but significant type-specific variations were noted. EV-D68 was detected more frequently in specimens from AFM patients with onset in 2016 and EV-A71 more often in those with onset in 2018. These variations might have contributed to differences in clinical characteristics seen between peak years. However, removal of EV-A71–positive cases did not eliminate the differences in clinical characteristics between peak years, suggesting that the greater number of EV-D68–positive cases in 2016 contributed to the clinical variability. Distinct AFM clinical presentations have been observed for different enterovirus etiologies (*15*,*16*). AFM caused by EV-A71 has been associated with myoclonus, ataxia, weakness, and autonomic instability. In an isolated outbreak in Colorado in 2018, AFM cases associated with EV-A71 were clinically distinct from those not associated with EV-A71 (*15*). Of note, paralytic syndrome caused by poliovirus is classically characterized by lower extremity weakness of an asymmetric distribution and a preceding mild gastrointestinal illness, features less common among AFM cases in peak years (*16*).

Although multiple viruses are associated with AFM, growing evidence suggests that nonpolio enteroviruses and specifically EV-D68 are linked to the changes in AFM epidemiology that started in 2014 (*21*,*22*). Enteroviruses were the most common viruses in nasopharyngeal, oropharyngeal, or fecal specimens from confirmed AFM patients identified by CDC researchers, and EV-D68 was the most frequent enterovirus typed (*18*–*20*). Unlike most other viruses known to cause AFM, enteroviruses routinely circulate and can cause outbreaks during the late summer and early fall months in the United States in a pattern corresponding with the observed seasonal AFM peaks (*23*,*24*). Although the United States does not have active national enterovirus surveillance, the enterovirus cases reported in 2 passive laboratory-based reporting systems (the National Enterovirus Surveillance System and the National Respiratory and Enteric Virus Surveillance System) demonstrate the presence of an annual enterovirus season with variation in the enterovirus types circulating each year (*23*,*25*,*26*). Climate, level of immunity in the host population, and viral fitness probably influence which strains dominate each year (*27*). If >1 specific type of enterovirus causes AFM, differences in circulating types could account for changes in AFM epidemiology from year to year. EV-D68 might be one such type. Respiratory disease surveillance indicates that EV-D68 appears to have circulated in a biennial pattern since 2014, corresponding with trends in AFM. In 2014, 2016, and 2018, increases in respiratory disease caused by EV-D68 coincided with the increases in AFM (*24*,*28*–*30*). Since 2014, the correlation between EV-D68 circulation and AFM incidence has also been documented in Canada, Japan, Europe, and Argentina (*31*–*34*). Global collaborations for the investigation of AFM cases and ongoing, active enterovirus surveillance will enable a broader and more complete picture of enterovirus circulation patterns and their relationships to AFM in the future.

Sentinel surveillance of other enteroviruses, such as coxsackieviruses A2 and A4, have also demonstrated a biennial periodicity like that observed for EV-D68 (*35*), although neither of these coxsackieviruses have been implicated in clusters of AFM. Rotavirus has also been shown to have a biennial circulation pattern in the postvaccination era (*36*). These biennial circulation patterns might be caused by an increase in the number of young, unexposed persons during years of low circulation, which leads to a larger number of susceptible persons acquiring and transmitting the infection in the following year. However, this phenomenon cannot fully explain the periodicity seen with AFM. The median age of AFM patients in peak years (5 years) is higher than the median age of patients with respiratory EV-D68 infections (3 years) (*24*), possibly indicating that other factors besides viral infection affect the risk for AFM development. Moreover, limited data show that persons across all age groups have robust neutralizing antibody titers against EV-D68 (*37*), including against both historical and contemporary outbreak strains, implying ongoing exposure and infection across the United States. The development of AFM in a small percentage of patients infected by this ubiquitous virus is likely to depend on other factors. Research into environmental or genetic risk factors for AFM development will provide insight into AFM pathogenesis.

Our findings are subject to limitations. First, differences in types of sterile and nonsterile specimens collected and sent to the CDC during 2015–2018 might have affected comparisons of enterovirus/rhinovirus positivity of cases in different years. However, because all enterovirus/rhinovirus-positive specimens were analyzed for enterovirus type, the percentage of type-specific (e.g., EV-D68 or EV-A71) cases among enterovirus/rhinovirus-positive cases would not have been affected. Second, we considered specimens enterovirus- or rhinovirus-positive only if the CDC laboratory confirmed this finding. Although CDC staff requested specimens for testing and confirmation, they might not have received all of them, thus influencing the results reported here. Last, the reporting of suspected cases to CDC public health staff is inconsistent, despite efforts to increase healthcare provider recognition of AFM. Year-to-year variation in reporting can occur, and more comprehensive reporting by healthcare providers might occur during peak years, when their awareness of this illness is heightened.

The alternate-year pattern in peak AFM activity since 2014 highlights a noteworthy shift in the epidemiology of this syndrome. Differences between AFM cases in peak years and nonpeak years provide additional evidence to support the hypothesis of a unique pathogen or pathogens contributing to this new epidemiology. Multiple lines of evidence support EV-D68 as a leading candidate, although additional research is needed. Frequent detection of EV-A71 in AFM cases in 2018 illustrates that >1 virus can cause outbreaks of AFM, and therefore AFM surveillance should not be restricted to detection of a specific pathogen. Healthcare providers thoroughly documenting clinical findings, including results of complete neurologic examinations, and reporting AFM cases to public health authorities, regardless of the pathogen implicated by test results, have implications for treatment and prevention. National AFM surveillance data can be used to characterize yearly variations in AFM cases (temporally, clinically, and etiologically) and illuminate the pathology of this emerging illness.
